# Tumor Suppressor Gene Inactivation during Cadmium-Induced Malignant Transformation of Human Prostate Cells Correlates with Overexpression of *de Novo* DNA Methyltransferase

**DOI:** 10.1289/ehp.10207

**Published:** 2007-07-19

**Authors:** Lamia Benbrahim-Tallaa, Robert A. Waterland, Anna L. Dill, Mukta M. Webber, Michael P. Waalkes

**Affiliations:** 1 Inorganic Carcinogenesis Section, Laboratory of Comparative Carcinogenesis, National Cancer Institute at the National Institute of Environmental Health Sciences, National Institutes of Health, Department of Health and Human Services, Research Triangle Park, North Carolina, USA; 2 Departments of Pediatrics and Molecular and Human Genetics, Baylor College of Medicine, Children’s Nutrition Research Center, Houston, Texas, USA; 3 Department of Medicine and Department of Zoology, Michigan State University, East Lansing, Michigan, USA

**Keywords:** cadmium, carcinogenesis, DNA methylation, DNMT3b, p16, prostate, RASSF1A

## Abstract

**Background:**

Aberrant DNA methylation is common in carcinogenesis. The typical pattern appears to involve reduced expression of maintenance DNA methyltransferase, *DNMT1*, inducing genomic hypomethylation, whereas increased expression of *de novo DNMT3a* or *3b* causes gene-specific hypermethylation.

**Objectives:**

During cadmium-induced malignant transformation, an unusual pattern of genomic hypermethylation occurred that we studied to provide insight into the roles of specific DNMTs in oncogenesis.

**Methods:**

Gene expression and DNA methylation were assessed in control and chronic cadmium-transformed prostate epithelial cells (CTPE) using reverse transcription–polymerase chain reaction (RT-PCR), Western blot analysis, methylation-specific PCR, and methyl acceptance assay.

**Results:**

During the 10-weeks of cadmium exposure that induced malignant transformation, progressive increases in generalized DNMT enzymatic activity occurred that were associated with over-expression of *DNMT3b* without changes in *DNMT1* expression. Increased *DNMT3b* expression preceded increased DNMT enzymatic activity. Procainamide, a specific DNMT1 inhibitor, reversed cadmium-induced genomic DNA hypermethylation. Reduced expression of the tumor suppressor genes, *RASSF1A* and *p16*, began about the time *DNMT3b* overexpression first occurred and progressively decreased thereafter. *RASSF1A* and *p16* promoter regions were heavily methylated in CTPE cells, indicating silencing by hypermethylation, while the DNA demethylating agent, 5-aza-2′-deoxycytidine, reversed this silencing. DNMT1 inhibition only modestly increased *RASSF1A* and *p16* expression in CTPE cells and did not completely reverse silencing.

**Conclusions:**

These data indicate that *DNMT3b* overexpression can result in generalized DNA hypermethylation and gene silencing but that DNMT1 is required to maintain these effects. The pattern of genomic DNA hypermethylation together with up-regulation of *DNMT3b* may provide a unique set of biomarkers to specifically identify cadmium-induced human prostate cancers.

In mammals the methylation of cytosine residues in DNA by DNA methyltransferases (DNMTs) is the predominant postreplication base modification (Das and Singal 2004). Mammalian DNMTs transfer a methyl group from *S*-adenosylmethionine (SAM) to the C-5 position of cytosine within CpG dinucleotides (Das and Singal 2004). DNMTs found in mammalian cells include DNMT1, DNMT3a, and DNMT3b (Okano et al. 1998, 1999a). It is thought that DNMTs function in either maintenance of DNA methylation, where methylated CpG sites on one DNA strand are copied, or in *de novo* methylation, where both strands are initially unmethylated and methylation at novel sites is introduced (Das and Singal 2004). Specific forms of DNMT include *DNMT1*, which is considered important for maintenance and is constitutively expressed in adult cells, and *DNMT3a* and *3b*, which have strong *de novo* activity (Das and Singal 2004) and are highly expressed in embryonic cells (Okano et al. 1999b), presumably to assist in methylation changes associated with development. Defects in methylation of total DNA or particular DNA sequences, including hypomethylation and, conversely, hypermethylation, have been shown to be associated with carcinogenesis, possibly as a factor that facilitates aberrant under- or over-expression of genes linked to cancer (Das and Singal 2004). Aberrant methylation of promoter regions resulting in inactivation of human tumor suppressor gene expression has been proposed to be an important mechanism in cancer progression (Baylin et al. 1991). Understanding what modulates changes in DNA methylation during malignant transformation is a key issue in chemical carcinogenesis. In this regard, during carcinogenesis, global DNA hypomethylation together with gene-specific hypermethylation, often, but not always, occur. It is suspected that *DNMT1* underexpression causes the total DNA hypomethylation, whereas over-expression of *DNMT3a* or *3b* induces gene-specific promoter region hypermethylation and quiescence of tumor suppressor genes (Baylin et al. 1991; Chen et al. 2003). For example, hypermethylation in the promoter region and silencing of *p16* tumor suppressor gene are frequently observed during carcinogenesis that can occur on a background of global DNA hypomethylation (Wu et al. 2005). Cancer cells may be distinctive in that DNMT1 alone is not responsible for maintaining abnormal gene-specific hypermethylation, and both DNMTs 1 and 3b may cooperate in this action (Rhee et al. 2000, 2002). However, the precise events that account for changes in global or gene-specific methylation patterns in carcinogenesis remain uncertain.

Methylation changes occur during oncogenic transformation with inorganic carcinogens such as arsenic, nickel, and cadmium (Benbrahim-Tallaa et al. 2005; Salnikov and Costa 2000; Takiguchi et al. 2003). For cadmium, a heavy metal classified as a human carcinogen, human exposure is associated with lung and possibly prostate cancer, whereas in rodents the metal is clearly a prostate carcinogen (Waalkes 2003). In *in vitro* model systems of carcinogenesis, cadmium exposure malignantly transforms various human and rodent cells, which give rise to aggressive cancers when injected into mice (Achanzar et al. 2001; Qu et al. 2005; Waalkes 2003). In particular, cadmium induces malignant transformation of the normal human prostate epithelial cell line RWPE-1 (Achanzar et al. 2001), which supports the potential for cadmium to directly target this cell population *in vivo*. In this prior work (Achanzar et al. 2001), to achieve malignant transformation, control human prostate epithelial RWPE-1 cells were exposed continuously to 10 μM cadmium for 8 or more weeks, a concentration near the estimated range in the prostate of adult human males with no known occupational exposure to cadmium. The cadmium-treated cells, designated CTPE (cadmium-transformed prostate epithelial cells), form malignant tumors after inoculation into nude mice that morphologically and biochemically resemble human prostatic carcinoma (Achanzar et al. 2001). Thus, the cadmium-transformed CTPE cells provide a human model system in which to examine the molecular events during cadmium carcinogenesis in the prostate.

Previous work showed an unusual pattern of enhanced generalized DNMT activity and global DNA hypermethylation during cadmium induction of malignant transformation in rat liver epithelial cells (Takiguchi et al. 2003). Thus, in the present study, we assessed the impact of chronic cadmium exposure leading to acquisition of a malignant phenotype on DNA methylation and DNMT activity in a human cell model using CTPE cells.

## Materials and Methods

### Chemicals and reagents

Cadmium chloride (CdCl_2_; purity 99%), 5-aza-2′-deoxycytidine, and procainamide were purchased from Sigma Chemical Co. (St. Louis, MO). Keratinocyte serum-free medium (K-SFM), epidermal growth factor (EGF), bovine pituitary extract (BPE), 100 X antibiotic–antimycotic mixture, and TRIzol reagent were purchased from Life Technologies, Inc. (Grand Island, NY). The mouse anti-p16 was purchased from (Calbiochem, San Diego, CA). The mouse anti-RASSF1A was purchased from (Abcam, Cambridge, MA). The mouse monoclonal anti-actin was purchased from (Oncogene Research Products, Cambridge, MA). The Bradford Protein Assay was purchased from Bio-Rad Laboratories (Hercules, CA).

### Cell culture and treatment

The non-tumorigenic control (untransformed) RWPE-1 cells were originally derived from immortalized normal human prostate epithelial cells and were grown as previously described (Achanzar et al. 2001). Cells were exposed continuously to 10 μM cadmium chloride for up to 10 weeks. The cadmium-transformed cells were designated CTPE cells to distinguish them from the control (untreated) RWPE-1 cells. Parallel cultures grown in cadmium-free medium provided passage-matched controls. CTPE cells produced aggressive carcinomas after inoculation into nude mice, with morphologic and molecular characteristics of prostate epithelial carcinoma including prostate-specific antigen (PSA) overexpression (Achanzar et al. 2001). Control RWPE-1 cells did not produce tumors upon inoculation (Achanzar et al. 2001).

To evaluate *p16* re-expression, we added the demethylating agent 5-aza-2′-deoxycytidine (0, 0.5, or 1 μM) to 50% confluent cell cultures. The cultures were dosed every 48 hr and harvested on day 6 for isolation of proteins. Alternatively, in a time-course experiment, 50% confluent cells were exposed to 1 μM 5-aza-2′-deoxycytidine for 0–6 hr. Procainamide (0, 0.5, or 1 mM) was added to 50% confluent cell cultures. The cultures were dosed every 48 hr, then harvested after 96 hr for isolation of proteins.

### Global DNA methylation and DNMT activity

The extent of global DNA methylation was determined by the methyl acceptance using [^3^H]SAM and bacterial Sss1 DNA methylase as previously described (Herman et al. 1996). DNMT activity of cell lysates was measured using the poly(dI.dC) poly(dI.dC) double-stranded DNA as the substrate and radiolabeled SAM as previously described (Jeronimo et al. 2004).

### RNA extraction and reverse transcription–polymerase chain reaction (RT-PCR)

Total RNA was isolated using TRIzol reagent (Life Technologies, Grand Island, NY). RT–PCR was performed using a TITANIUM One-Step RT-PCR kit (Clontech, San Jose, CA) and a GeneAmp PCR system 9700 (Applied Biosystems, Foster City, CA) according to the kit’s instructions. Amplification conditions were 60 min at 50°C and 5 min at 94°C followed by 35 cycles for 1 min at 94°C, 1 min at 57°C, and 1 min at 72°C; 1 μg of total RNA was used in each amplification. Primers were designed for DNMT1, DNMT3a, DNMT3b, MBD2, and β-actin and were synthesized by Invitrogen (Carlsbad, CA) as follows: DNMT1 (gi: 6684524) (5′-ACCAAGCAAGAAGTGAAGCC-3′ and 5′-GCTTCCTGCAGAAGAACCTG-3′), product size: 366 bp; DNMT3a (gi:18033252) (5′-CACACAGAAGCATATCCAGGAGTG-3′ and 5′-AGTGACTGGGAAACCAAATACCC-3′), product size: 551 bp; DNMT3b (gi: 18033254) (5′-AATGT-GAATCCAGCCAGGAAAGGC-3′and 5′-ACTGGATTACACTCCAGGAACCGT-3′) product size: 190 bp; β-actin (5′-AGAGATGGCCACGGCTGCTT-3′ and 5′-ATTTGCGGTGGACGATGGAG-3′), product size: 460 bp. The level of β-actin was used to normalize results.

### Real-time RT-PCR analysis

Expression levels of selected genes were quantified using real-time RT-PCR analysis. In brief, total RNA was extracted as described above, purified with RNeasy column and on-column DNase I digestion (QIAGEN, Valencia, CA). Purified total RNA was reverse-transcribed with MuLV reverse transcriptase and oligo-dT primers. The forward and reverse primers were synthetized by Invitrogen as fellows: *DNMT1* (5′-GGTTCTTCCTCCTGGAGAATGTC-3′ and 5′-GGGCCACGCCGTACTG-3′); *DNMT3a* (5′-AGGAAGCGCAAGCACCC-3′ and 5′-ATTGGGTAATAGCTCTGAGGCG-3′); *DNMT3b* (5′-CCAGCCCTGCGGCAG-3′ and 5′-GTTGACGAGGATCGAGTCTTCC-3′) and *18S* (gi: 337376) (5′-CGTTGATTAAGTCCCTGCCCTT-3′ and 5′-TCAAGTTCGACCGTCTTCTCAG-3′). Quantitative assessments of DNA amplification for each gene were performed by a fluorescence-based real-time detection method (Bio-Rad Laboratories GmbH, Munchen, Germany) and the iQ SYBR Green Supermix. The dissociation curve (melting curve) for each gene was performed to verify the quality of the primers. The standard curve for each gene was performed to quantify the gene expression. Gene expression was first normalized relative to 18S RNA within the same sample, and then treatment group expression was calculated by setting the control as 100%.

### Western blot analysis

Total protein was isolated and electrophoresed on NuPAGE 4–12% Bis-Tris gel and transferred to nitro-cellulose membranes according to the manufacturer’s directions (Invitrogen). Immunoblotting was performed using the human p16 antibody (Calbiochem), the human RASSF1A antibody (Abcam), and horseradish peroxidase-conjugated anti-mouse secondary antibody (Amersham, Piscataway, NJ).

### Promoter region methylation of RASSF1A and p16

DNA methylation in the CpG islands of the *p16* promoter region was determined by methylation-specific PCR (MSP). MSP distinguishes unmethylated from methylated alleles on the basis of sequence changes produced after bisulfite treatment of DNA, which converts unmethyled cytosine to uracils, and subsequent PCR using primers designed for either methylated or unmethylated DNA (Balaghi and Wagner 1993). The primers used were as follows: unmethylated reaction 5′-TTTGGTTGGAGTGTGTTAATGTG-3′ (sense) and 5′-CAAACCCCACAAACTAAAAACAA-3′ (antisense) and for the methylated reaction 5′-GTGTTAACGCGTTGCGTATC-3′ (sense) and 5′-AACCCCGCGAACTAAAAACGA-3′ (anti-sense) for *RASSF1A* and 5′-TTATTAGAGGGTGGGGTGGATTGT-3′ (sense) and 5′-CCACCTAAATCAACCTCCAACCA-3′ (antisense) and for the methylated reaction 5′-TTATTAGAGGGTGGGGCGGATCGC -3′ (sense) and 5′-CCACCTAAATCGACCTCCGACCG-3′ (antisense) for *p16*.

### Statistical analysis

All data represent mean ± SE derived from three or more independent experiments. Statistical significance was determined by the Student *t*-test or by analysis of variance followed by Dunnett’s multiple comparison test as appropriate. A *p* ≤ 0.05 was considered statistically significant in all cases.

## Results

### DNA methyltransferases activity and expression and total DNA methylation during cadmium-induced malignant transformation

**Figure 1:**
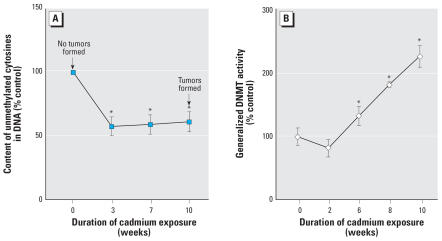
Global DNA methylation status and DNA methyltransferase activity during cadmium transformation of human prostate epithelial cells. Cells were grown in the presence or absence of 10 μM of cadmium for up to 10 weeks. (*A*) DNA methylation in control and exposed cells was determined by the *in vitro* methyl acceptance capacity of DNA using [^3^H-methyl]SAM as a methyl donor and a prokaryotic CpG DNA methyltransferase as described in “Materials and Methods.” Arrows indicate the ability of cells to form tumors upon inoculation into Nude mice according our prior work (Achanzar et al. 2001). (*B*) DNA methyltransferase activity was assayed as described in “Materials and Methods.” Data represent mean ± SE (*n* = 3). *Significantly different from control.

Chronic cadmium exposure of human prostate epithelial cells led to a significant increase in global DNA methylation concurrently with transformation, as evidenced by a 53% decrease in unmethylated sites in DNA in the CTPE cells. Increased methylation occurred prior to cadmium-induced transformation (Figure 1A), clearly indicating that DNA methylation errors occurred during the process of cadmium-induced malignant transformation.

**Figure 2:**
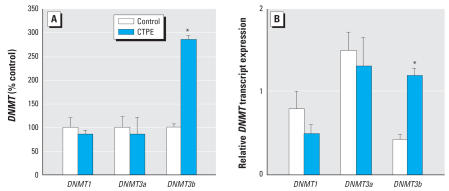
*DNMT* isoform transcript levels during cadmium exposure in CTPE cells. Transcript levels of specific forms of DNMT were assessed in control and CTPE cells exposed for 10 weeks to cadmium. RNA was isolated and subjected to RT-PCR analysis using a set of primers designed to amplify *DNMT1, DNMT3a, DNMT3b*, and β*-actin* transcripts. PCR products were separated on a 1.7% agarose gel. Densitometric data are given as percent of control (*A*) and normalized to β-actin (*B*) and expressed as mean ± SE (*n* = 3). *Significantly different from control.

Generalized DNMT enzymatic activity also exhibited a remarkable increase during cadmium-induced malignant transformation (Figure 1B). Enzymatic activity of DNMT increased up to 220% in CTPE cells compared with control cells. Transcript levels of the various *DNMT* isoforms showed no significant changes in CTPE cells with the single exception of the *de novo DNMT3b*, which increased nearly 3-fold (Figure 2).

**Figure 3:**
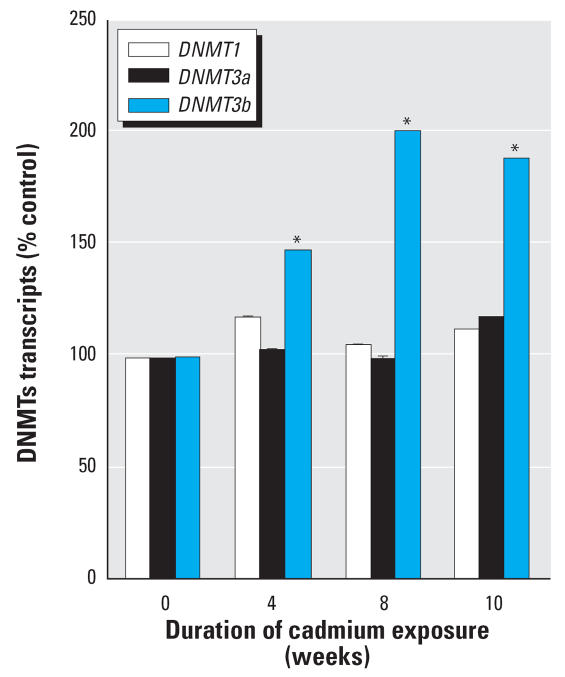
Analysis of the effect of chronic cadmium exposure of prostate epithelial cells on *DNMT3b* and *DNMT1* mRNA expression. Cells were grown in the presence or absence of 10 μM cadmium for up to 10 weeks. RNA was isolated and subjected to real-time RT-PCR analysis using a set of primers designed to amplify *DNMT3b*, *DNMT1,* and *18S* gene products. PCR products were separated on a 1.7% agarose gel. Densitometric data are given as percent of control and expressed as mean ± SE (*n* = 3). *Significantly different from control.

The time course for *DNMT3b* expression showed a gradual, time-dependent, progressive increase during cadmium transformation (Figure 3). The increased expression of *DNMT3b* was significant at 4 weeks of cadmium exposure. A similar time-course assessment for *DNMT1*and *DNMT3a* expression indicated that no significant changes occurred during cadmium-induced acquisition of malignant phenotype.

### Expression and promoter methylation of tumor suppressor genes RASSF1A and p16 during cadmium-induced malignant transformation

**Figure 4:**
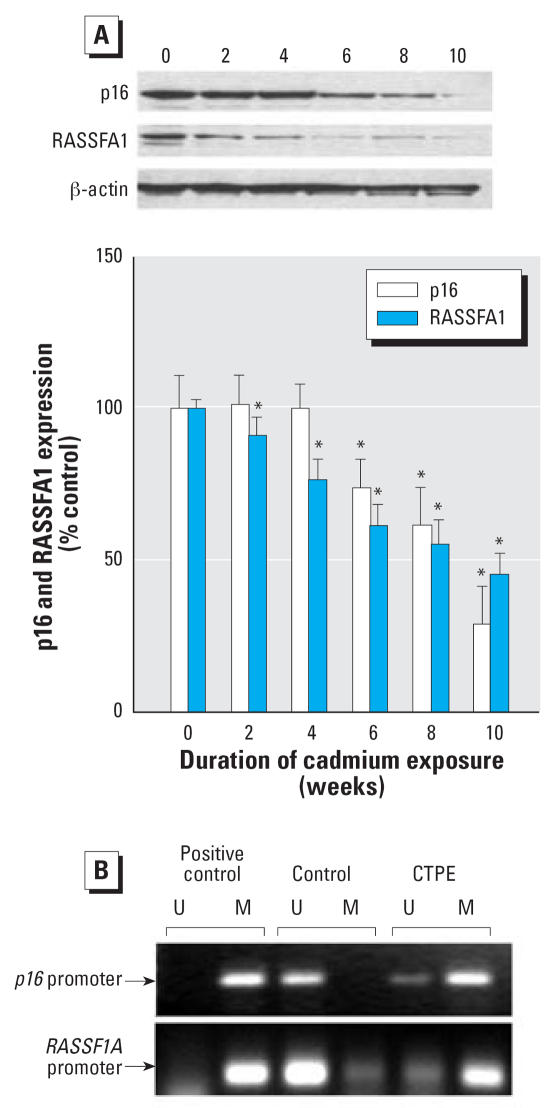
Expression and promoter region methylation status of *p16* and *RASSF1A* during chronic cadmium exposure. Cells were grown in the presence or absence of 10 μM cadmium for up to 10 weeks. (*A*) Proteins were isolated and subjected to Western blot analysis using antibodies against p16, RASSF1A, and β-actin. Blots were analyzed densitometrically, normalized to β-actin, and expressed as percent of control. (*B*) Methylation of the *p16* and *RASSF1A* promoter regions determined by MSP. The presence of visible PCR product in lanes marked “U” indicates the unmethylated genes, whereas the presence of product in lanes marked “M” indicates the methylated gene. The source of DNA is indicated above each lane. CpGENOME universal methylated DNA (CHEMICON International, Inc., Temecula, CA) was used as a positive control.

The expression of tumor suppressor genes *RASSF1A* and *p16* was progressively reduced during cadmium exposure (Figure 4A). The inhibition of *RASSF1A* expression was an early event that started by 2 weeks of cadmium exposure, whereas the inhibition of *p16* started by 6 weeks of cadmium exposure, which is soon after DNMT3b expression increases became significant (Figure 3).

**Figure 5:**
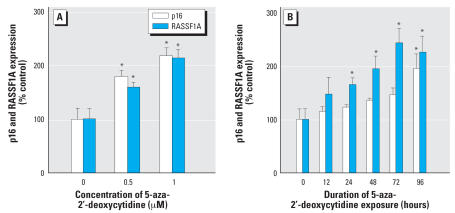
Effect of a demethylating agent 5-aza-2′-deoxycytydine on p16 and RASSF1A protein expression. (*A*) CTPE cells were grown in the presence or absence of 0.5 or 1 μM of 5-aza-2′-deoxycytydine for 6 days. (*B*) CTPE cells were grown in presence or absence of 1 μM of 5-aza-2′-deoxycytydine over 96 hr. Proteins were isolated and subjected to Western blot analysis using antibodies against p16, RASSF1A and β-actin. Blots were analyzed densitometrically, normalized to β-actin, and expressed as percent of control. Data represent mean ± SE (*n* = 3). *Significantly different from control.

To study the mechanism of cadmium-induced RASSF1A and p16 quiescence in CTPE cells, we assessed promoter region methylation. CpG islands in the RASSF1A and p16 promoter were clearly hypermethylated compared with control in CTPE cells (Figure 4B). This hypermethylation likely accounts for cadmium-induced silencing of these important tumor suppressor genes. In fact, when CTPE cells were treated with the DNA demethylating agent, 5-aza-2′-deoxycytidine, it effectively reversed the loss of RASSF1A and p16 expression that occurred during cadmium transformation (Fig. 5A). Time-course analysis indicated that 5-aza-2′-deoxycytidine (1 μM) increased RASSF1A protein expression nearly 2.4-fold (*p* < 0.05) and p16 about 2-fold (*p* < 0.05) after 96 hr of treatment (Figure 5B).

### Effect of procainamide on global DNA methylation and RASSF1A and p16 expression in control and CTPE cells

**Figure 6:**
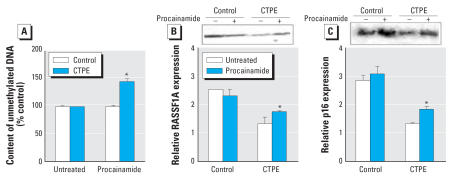
DNA methylation status, p16 and RASSFA1 protein expression after exposure to procainamide in CTPE cells. CTPE cells were grown in the presence or absence of 0.5 mM procainamide over 96 hr. (*A*) DNA methylation in control and CTPE cells was determined by the *in vitro* methyl acceptance capacity of DNA using [^3^H-methyl]SAM as a methyl donor and a prokaryotic CpG DNA methyltransferase as described in “Materials and Methods.” Proteins were isolated and subjected to Western blot analysis using antibodies against (*B*) RASSF1A and p16, and β-actin. Blots were analyzed densitometrically and normalized to β-actin. Data represent mean ± SE (*n* = 3). *Significantly different from control.

The treatment with procainamide, a specific DNMT1 inhibitor, caused a clear increase in unmethylated DNA content to above control levels, indicating that DNMT1 plays a key role in maintaining the aberrant genomic methylation pattern acquired during cadmium-induced malignant transformation in human prostate epithelial cells (Figure 6A).

DNMT1 inhibition only modestly increased both RASSF1A (23%) (Figure 6B) and p16 (28%) (Figure 6C) expression at the protein level in CTPE cells. Although procainamide induced increases in RASSF1A and p16 expression in CTPE cells, their expression was still well below control levels, indicating that DNMT1 is only partially responsible for maintaining gene-specific promoter region hypermethylation.

## Discussion

The present work indicates that DNA hypermethylation at the global and gene-specific levels occurred in association with cadmium-induced malignant transformation of human prostate epithelial cells. This total and gene-specific hypermethylation is associated with an overexpression of *DNMT3b* without changes in *DNMT1* expression. Increased expression of *DNMT3b* in CTPE cells occurred as an early event in response to chronic cadmium exposure, and, importantly, in advance of malignant transformation. Exactly how cadmium leads to the transcriptional up-regulation of the *DNMT3b* gene is unclear. The *de novo* DNMTs, such as 3b, are thought to be critical in tumor suppressor gene silencing by promoter region hypermethylation. For example, both *RASSF1A* and *p16* are frequently inactivated in human malignancies by methylation changes during malignant conversion (Auerkari 2006; Baylin et al. 1991; Kuzmin et al. 2002; Perry et al. 2006; Wu et al. 2005). Thus, the increase in *DNMT3b* expression seen in CTPE cells likely accounts for the progressive silencing of *RASSF1A* and *p16*, which was reversible after treatment with a general DNA demethylating agent. Our data also indicated that DNMT1 was critical in maintaining this aberrant total DNA methylation level, but probably had much less impact on gene-specific hypermethylation. Similarly, rat liver cells chronically treated with cadmium show global DNA hypermethylation and increased overall DNMT enzyme activity during malignant transformation (Takiguchi et al. 2003). Hence, the occurrence of global DNA hypermethylation appears to be a consistent finding in cadmium-induced malignant conversion. Thus, the deregulation of *DNMT3b* in CTPE cells occurred before the characteristics of malignant transformation were detectable and may account for the acquisition of malignant phenotype as well as the silencing of the tumor suppressor genes, *RASSF1A* and *p16*.

The exact mechanism behind oncogenic changes in locus-specific and global DNA methylation in human cells is unclear. *DNMT1* expression showed no changes during cadmium transformation. However, our data indicates DNMT1 activity was critical to the maintenance of cadmium-induced aberrant global DNA hypermethylation. Human cancer cells differ in their reliance on DNMT1 for maintaining DNA methylation (Rhee et al. 2000a; Ting et al. 2006), but it is clearly required to maintain the aberrant DNA methylation in CTPE cells. Treatment with procainamide, a specific DNMT1 inhibitor, completely reversed cadmium-induced global DNA hypermethylation. DNMT1 inhibition did not, however fully restore the cadmium-induced loss of RASSF1A and p16 expression, indicating that DNMT1 is only partially required for maintaining locus-specific changes in methylation in CTPE cells. Thus DNMT1, although not overexpressed, apparently acts in cooperation with DNMT3b. This is consistent with what is observed in human colorectal carcinoma cells, which appear to require both DNMT1 and DNMT3b expression to retain *p16* silencing via promoter region hypermethylation (Baylin et al. 1998). In addition, over-expression of DNMT3b, but not DNMT1 and DNMT3a, is common in various cancer cells (Girault et al. 2003; Robertson et al. 1999; Saito et al. 2002), suggesting that DNMT3b plays an important role in the development of aberrant promoter methylation during oncogenesis. Thus, even though DNMT1 expression is unaltered in CTPE cells, it appears to be required for maintenance of aberrant global DNA hypermethylation, and, to a lesser extent, at the locus-specific level.

Several studies indicate that DNA hypermethylation is an important mechanism in prostate cancer initiation and progression. Studies indicate more than 30 genes can undergo aberrant hypermethylation in prostate cancer (Li et al. 2004; 2005). These include tumor-suppressor genes, such as *RASSF1A* and *p16*, as well as genes involved in a number of key cellular pathways like hormonal response, cell cycle control, and tumor-cell invasion (Jeronimo et al. 2004). For many of these genes, promoter hypermethylation is often the main mechanism underlying their functional loss in prostate cancer (Jeronimo et al. 2004; Li et al. 2004, 2005). Indeed *RASSF1A* and *p16* silencing in CTPE cells was reversed by the DNA demethylating pyrimidine analog 5-aza-2′-deoxycytidine in the present study. Inappropriate silencing of genes in prostatic epithelial cells may contribute to all stages of cancer development, including initiation, progression, invasion, and metastasis. In fact, hypermethylation-based gene silencing has been correlated with acquisition of androgen independence in advanced prostate cancer (Jeronimo et al. 2004). Tumors showing acquired androgen independence are usually much more aggressive and frequently fatal (Jeronimo et al. 2004). Our work also indicates that during cadmium-induced transformation, CTPE cells acquire androgen independence (Benbrahim-Tallaa et al. 2007), and the propensity for cadmium to induce DNA hypermethylation may contribute to this conversion. An insidious but common environmental pollutant like cadmium that could potentially drive human prostate cancer progression to a more lethal phenotype deserves further study.

*DNMT3b* is frequently overexpressed in tumor cells and its expression has been correlated with decreasing differentiation (Robertson et al. 1999). It is suspected that DNMT3b is at least partially responsible for the aberrant methylation observed in cancer cells and that it is required for the active suppression of genes (Beaulieu et al. 2002; Jin et al. 2005; Lin et al. 2005). However, although DNMT3b is generally considered a *de novo* DNMT that can methylate DNA at novel sites, it may also be critical for the maintenance of global DNA methylation (Beaulieu et al. 2002; Chen et al. 2003; Jin et al. 2005; Lin et al. 2005). The results of the present study indicate DNMT3b and DNMT1 cooperatively maintain cadmium-induced aberrant DNA methylation and gene silencing in malignantly transformed CTPE cells. However, defining the mechanisms of cadmium-induced *DNMT3b* overexpression and the precise role of DNMT3b in DNA hypermethylation will require further research.

In summary, the present work demonstrates that overexpression of *DNMT3b* was associated with cadmium-induced malignant transformation in human prostate epithelial cells and caused genomewide and gene-specific DNA hypermethylation in association with DNMT1. *DNMT3b* overexpression occurred contemporaneously with global DNA hypermethylation and tumor suppressor gene silencing through increased promoter region methylation. Finally, the pattern of genome-wide hypermethylation and *DNMT3b* over-expression combined with tissue cadmium levels may provide biomarkers to specifically identify cadmium-induced human prostate cancers.
